# Tools for *in vivo* labelling of ALFA-tagged proteins in *C. elegans* .

**DOI:** 10.17912/micropub.biology.002419

**Published:** 2026-10-21

**Authors:** Irini Topalidou, Nicolas Lehrbach

**Affiliations:** 1 Basic Sciences Division, Fred Hutchinson Cancer Center, Seattle, Washington, United States

## Abstract

Understanding protein function requires tools to visualize and manipulate proteins *in vivo* . NanoTags, short epitope tags that are recognized by single-chain antibodies, are an emerging method for versatile *in vivo* labelling and manipulation of endogenous proteins. The short (13 amino acid) ALFA tag has been shown to allow labelling of endogenous proteins in several model systems, including *C. elegans* . Here we describe the generation and validation of transgenes to facilitate global and tissue-specific visualization of ALFA-tagged endogenous proteins in *C. elegans* . We propose a modular NanoTag system as a powerful approach for investigating protein functions *in vivo* in *C. elegans* .

**Figure 1. Tools for in vivo labelling of ALFA-tagged proteins in C. elegans:** (A) Fluorescence images showing expression of NbALFA::GFP and labelling of ALFA-tagged endogenous proteins. Images of WT animals are included to show levels and localization of NbALFA::GFP in the absence of an ALFA-tagged target protein. ALFA-tagged nuclear proteins HIS-72 and LMN-1 cause increased nuclear localization of NbALFA::GFP. Expression of UNC-54 is restricted to the body wall muscle, so introduction of the tag does not alter NbALFA::GFP localization in non-muscle tissues (see panel (D) for a higher magnification image showing NbALFA::GFP-mediated labelling of UNC-54 ::ALFA in muscle cells). (B) Fluorescence images showing expression of NbALFA::tagRFP and labelling of ALFA-tagged nuclear lamin LMN-1 . (C) High magnification fluorescence images showing labelling of ALFA-tagged HIS-72 and LMN-1 using NbALFA::GFP. (D) High magnification image showing labelling of ALFA-tagged UNC-54 in the body wall muscle using NbALFA::GFP. An image of an animal in which UNC-54 is directly tagged with GFP is shown for comparison. NbALFA labelling is not as bright and is not accompanied by disruption of muscle fibers. (E) Fluorescence images showing tissue-specific labelling of ALFA-tagged HIS-72 with NbALFA::GFP.

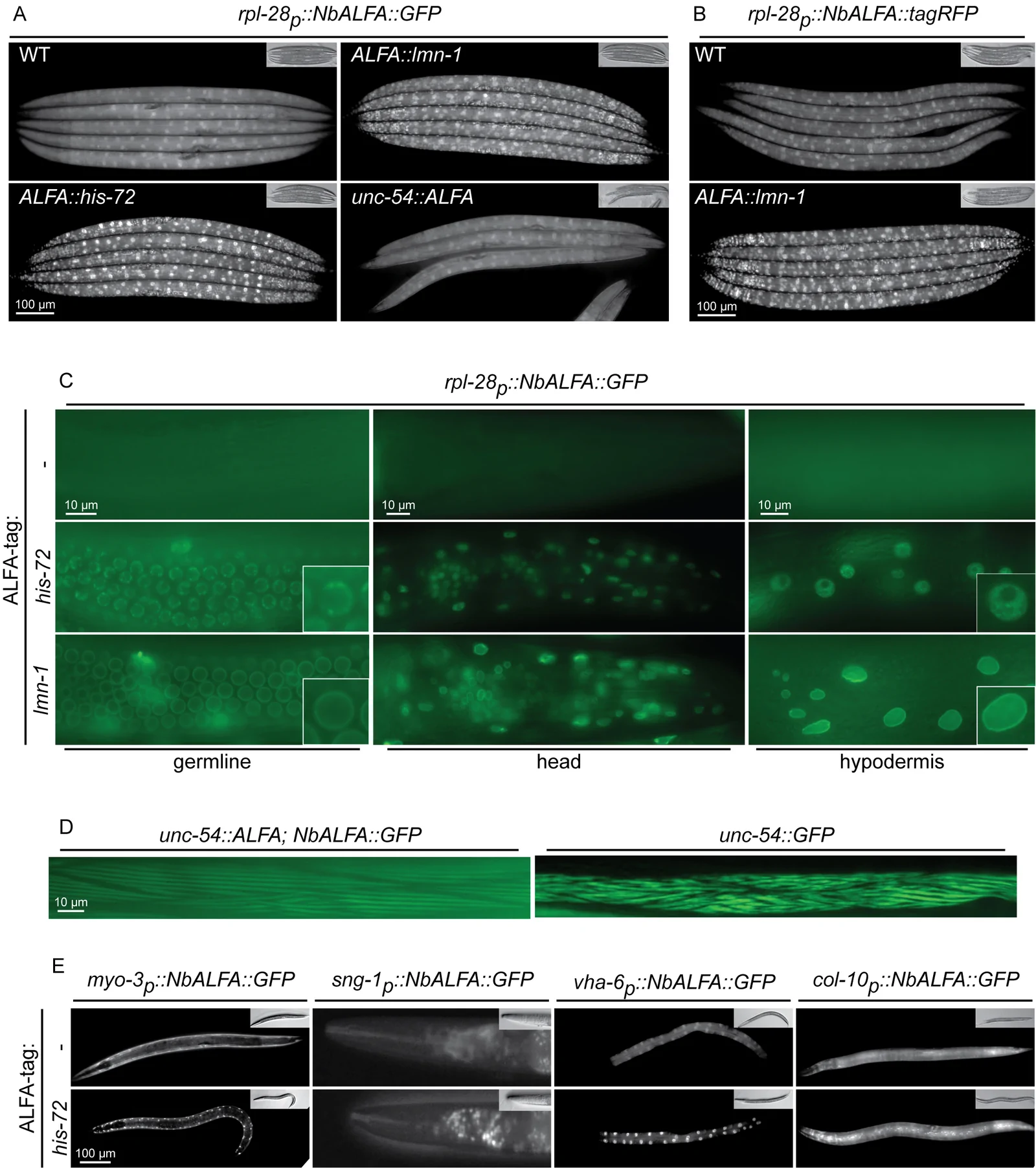



## Description

Studies of protein function rely on labelling tools to visualize or manipulate proteins *in vivo* . In genetically tractable model organisms such as *C. elegans* , CRISPR-mediated gene editing can be used to knock in fluorescent or other functionalized tags to endogenous proteins for visualization and/or manipulation (Artan et al., 2021; Ghanta & Mello, 2020; Kurashina & Mizumoto, 2023; Schwartz et al., 2021; Zhang et al., 2015). However, directly tagging proteins of interest is not ideal for all applications. Tags such as GFP or other fluorescent proteins are valuable for protein visualization by live microscopy. However, their relatively large size can interfere with protein structure or function. Further, directly tagging endogenous loci causes labelling of the protein of interest in all tissues where it is expressed, which can complicate analysis of tissue-specific protein functions. Finally, tagging a single protein with multiple fluorescent or other functional tags requires the generation and validation of a new edited line for each tag. Expanding the repertoire and modularity of tools for endogenous protein labelling would therefore accelerate research into protein function *in vivo* .

NanoTags, short epitope tags that are bound at high affinity by single-chain antibodies (nanobodies, or Nbs), offer several advantages for *in vivo* labelling of endogenous proteins (Cheloha et al., 2020). NanoTag epitopes are typically small (~10-15 amino acids), reducing the possibility that the tag interferes with protein folding or function. Nanobodies can readily be expressed as recombinant proteins via transgenes, a strategy that has been used to label tagged proteins in various experimental systems (Igreja et al., 2022; Kim et al., 2022; Neveu et al., 2025; Quintin et al., 2025; van der Salm et al., 2026; Virant et al., 2018; Wang et al., 2025; Xu et al., 2022; Zeghal et al., 2023). The 13 amino acid ALFA-tag is recognized with high specificity and affinity by an ALFA-specific nanobody (NbALFA) (Gotzke et al., 2019). CRISPR/Cas9-mediated knock-in of an ALFA-tag allows detection or labelling of endogenous proteins with NbALFA (Igreja et al., 2022; Neveu et al., 2025; Quintin et al., 2025; van der Salm et al., 2026; Wang et al., 2025; Xu et al., 2022). Transgenic strains that express NbALFA fusions in *C. elegans* germline have been produced by two labs (NbALFA::mKate2, Quintin et al., 2025; and NbALFA::HaloTag, van der Salm et al., 2026) and shown to enable live imaging of ALFA-tagged endogenous proteins in the germline and embryos. Here, we confirm the broad utility of NbALFA for labelling endogenous proteins, by using transgenes to drive whole-animal and tissue-specific expression of NbALFA fusion proteins.

We generated constructs in which an NbALFA::GFP or an NbALFA::tagRFP fusion is controlled by the promoter of the ubiquitously expressed *rpl-28* gene. We then generated single-copy insertion lines for each construct using miniMos (Frokjaer-Jensen et al., 2014). We observed diffuse nuclear and cytosolic fluorescence in NbALFA::GFP and NbALFA::tagRFP transgenic animals. To test the ability of the NbALFA fusions to recognize ALFA-tagged endogenous proteins, we used CRISPR/Cas9 to ALFA-tag highly expressed proteins with easily detectable localization patterns: *his-72* , encoding a histone H3.3, which is expressed in all tissues and localizes to chromatin in the nucleus; *lmn-1* , encoding a nuclear lamin; and *unc-54* , which encodes myosin heavy chain (MHC B) expressed specifically in body wall muscle. In each case, we observed localization patterns consistent with specific labelling of the endogenously tagged protein ( Fig. 1A- D).

We noticed a slight increase in the overall fluorescence of NbALFA::GFP and NbALFA::tagRFP following ALFA-tagging of abundant endogenous proteins ( Fig. 1A- C). This appears to be a specific effect of the interaction between the NbALFA fusion and its target protein: UNC-54 is expressed specifically in muscle, and we did not observe a change in NbALFA::GFP levels in non-muscle cells of *unc-54* ::ALFA animals ( Fig. 1A ). These observations suggest that NbALFA fusion proteins are unstable when they are not bound to a target ALFA-tagged protein. A similar effect has been observed in mammalian cells, suggesting that NbALFA is generally unstable in animal cells unless bound to an ALFA-tagged target protein (Wang et al., 2025).

Labelling of endogenous proteins with fluorescent tags can, in some cases, interfere with the function of the tagged protein. GFP-tagging of LMN-1 and of the muscle myosin UNC-54 both cause sluggish locomotion, suggesting impaired function of the tagged protein (Arribere et al., 2016; Gregory et al., 2023). However, we did not observe any obvious change in locomotion of animals in which LMN-1 ::ALFA or UNC-54 ::ALFA is labelled by NbALFA::GFP. The organization of muscle filaments appears disrupted in *unc-54 ::gfp* animals but not in animals in which UNC-54 ::ALFA is labelled via NbALFA::GFP ( Fig. 1D ). These observations support the conclusion that ALFA-tagging provides a means to tag and visualize the localization of endogenous proteins by live imaging without the drawbacks of direct addition of a bulky fluorescent tag that can interfere with protein structure or function.

Finally, we sought to test the possibility of using NbALFA fusion proteins to label endogenous proteins in a tissue-specific manner. We generated transgenic animals expressing NbALFA::GFP under the control of several tissue-specific promoters and tested their ability to label HIS-72 ::ALFA in the corresponding tissues. In each case, we detected the expected increase in nuclear localization of NbALFA::GFP, indicating tissue-specific labelling of histone H3 ( Fig. 1E ). In addition to labelling of HIS-72 in the nucleus, we observed additional cytosolic signal in some tissues, which was especially pronounced with hypodermal *col-10 p* ::NbALFA::GFP. It is possible that strong expression of tissue-specific promoters leads to increased background signal from NbALFA::GFP that is not bound to the ALFA-tagged target.

Here, we have generated transgenic strains expressing NbALFA fused to fluorescent proteins to facilitate global and tissue-specific fluorescent labelling using NanoTagging in *C. elegans* . Our work provides new ways to achieve labelling in entire animals or in specific somatic tissues, which complements tools for labelling ALFA-tagged proteins in *C. elegans* already generated by other groups (Quintin et al., 2025; van der Salm et al., 2026). Importantly, van der Salm and colleagues' work demonstrates the use of orthogonal labelling strategies in combination with the ALFA tag for simultaneous live imaging of multiple proteins and discusses the advantages of indirect labelling in detail (van der Salm et al., 2026). We note that the transgenes we generated are randomly inserted into the genome via miniMos and we have not systematically determined their integration sites. Insertions of similar transgenes for tissue-specific NbALFA labelling at defined safe-harbor loci would improve their utility.

Because the short ALFA-tag is straightforward to introduce via CRISPR/Cas9, these tools can be used to quickly generate strains in which any protein of interest is labelled. We tested this system for labelling highly abundant proteins but have not yet carefully evaluated the extent of background signal (i.e. we do not know what fraction of fluorescent signal is derived from NbALFA::GFP/tagRFP molecules that are not bound to their target). This caveat of the ALFA-tagging approach contrasts with split-GFP labelling, in which the fluorophore is reconstituted upon binding to the target protein, yielding minimal background signal (Feng et al., 2019; He et al., 2019; Hefel & Smolikove, 2019; Noma et al., 2017). ALFA-tagging may be an attractive alternative to split-GFP in cases where background signal is not a concern, or where maximizing signal is important, since reconstituted split-GFP is much dimmer than intact GFP (Feng et al., 2019). Given the potential for background signal to complicate analysis, it will be important to test the ALFA system for detection of less abundant proteins and to explore strategies for increasing labelling specificity, perhaps by using NbALFA variants whose stability or fluorescence of the attached fluorophore is dependent on binding to an ALFA-tagged target (Barykina et al., 2026; Wang et al., 2025). Labelling efficiency could also be enhanced by incorporating multiple tags in tandem to a target protein, as van der Salm and colleagues demonstrate using the SunTag System.

Improving and expanding the NanoTag toolkit will enable new applications. For example, cell-type specific promoters would allow labelling of endogenous proteins with greater anatomical specificity. Fusion of NbALFA to other functional partners could be used to achieve various tissue-specific manipulations. Potential applications could include tissue-specific proximity labelling, manipulation of protein localization, protein-protein interactions, or stability. In conclusion, these tools and those reported by others (Igreja et al., 2022; Quintin et al., 2025; van der Salm et al., 2026) support the usefulness of NanoTags in *C. elegans* . We hope that continued development of NanoTagging tools for *C. elegans* will enable new approaches to investigating protein function *in vivo* .

## Methods

*C. elegans husbandry.*

*C. elegans* were maintained on standard nematode growth media (NGM) at 20°C and fed *E. coli* OP50 . Some strains were provided by the CGC, which is funded by NIH Office of Research Infrastructure Programs (P40 OD010440). The following strains were used in this study are listed below.

**Table:** 

Strain name	Genotype	Reference
NJL5098	*unc-119 ( kst33 )* III *; nicSi744 [ rpl-28 ::NbALFA::GFP:: tbb-2 ]* V	(Yanagi et al., 2026)
NJL5110	*unc-119 ( kst33 )* III *; nicSi749 [ rpl-28 p::NbALFA::tagRFP:: tbb-2 ] *	(Yanagi et al., 2026)
NJL5311	*lmn-1 ( nic1723 [ALFA])* I *; unc-119 ( kst33 )* III *; nicSi744 [ rpl-28 p::NbALFA::GFP:: tbb-2 ]* V	this study
NJL5313	*his-72 ( nic1725 [ALFA])* III *unc-119 ( kst33 )* III *; nicSi744 [ rpl-28 p::NbALFA::GFP:: tbb-2 ]* V	this study
NJL5319	*unc-54 ( nic1731 [ALFA])* I *; unc-119 ( kst33 )* III *; nicSi744 [ rpl-28 p::NbALFA::GFP:: tbb-2 ]* V	this study
NJL5312	*lmn-1 (nic1724[ALFA])* I *; unc-119 ( kst33 )* III *; nicSi749 [ rpl-28 p::NbALFA::tagRFP:: tbb-2 ] *	this study
NJL5334	*unc-119 ( kst33 )* III *; nicSi813 [ myo-3 p::NbALFA::GFP:: tbb-2 ] *	this study
NJL5327	*unc-119 ( kst33 )* III *; nicSi808 [ sng-1 p::NbALFA::GFP:: tbb-2 ] *	this study
NJL5351	*unc-119 ( kst33 )* III *; nicSi823 [ vha-6 p::NbALFA::GFP:: tbb-2 ] *	this study
NJL5392	*unc-119 ( kst33 )* III *; nicSi832 [ col-10 p::NbALFA::GFP:: tbb-2 ] *	this study
NJL5486	*his-72 ( nic1725 [ALFA]) unc-119 ( kst33 )* III *; nicSi813 [ myo-3 p::NbALFA::GFP:: tbb-2 ] *	this study
NJL5427	*his-72 ( nic1725 [ALFA]) unc-119 ( kst33 )* III *; nicSi808 [ sng-1 p::NbALFA::GFP:: tbb-2 ] *	this study
NJL5429	*his-72 ( nic1725 [ALFA]) unc-119 ( kst33 )* III *; nicSi823 [ vha-6 p::NbALFA::GFP:: tbb-2 ] *	this study
NJL5430	*his-72 ( nic1725 [ALFA]) unc-119 ( kst33 )* III *; nicSi832 [ col-10 p::NbALFA::GFP:: tbb-2 ] *	this study
PD2856	*unc-54 ( cc2856 [ unc-54 ::gfp])* I	(Arribere et al., 2016)

*Plasmid constructs and generation of transgenic animals.*

All cloning was carried out using the NEBuilder HiFi DNA Assembly kit (New England Biolabs #E2621). The sequence encoding NbALFA was codon-optimized for expression in *C. elegans* using the transgenebuilder tool (Beta Version; https://www.wormbuilder.org/transgenebuilder/) and synthesized to include a single intron (from the *rps-0* gene) and a C-terminal in-frame linker sequence (STSGGSGGTGGSS). Gene synthesis was carried out by Twist Biosciences. Gibson assembly was used to generate plasmids in which NbALFA fusion proteins are expressed under the control of various promoters: *rpl-28* promoter (ubiquitous expression, 605 bp immediately upstream of the *rpl-28* ORF), *myo-3* promoter (body wall muscle expression, 2139 bp immediately upstream of the *myo-3* ORF), *sng-1* promoter (neuronal expression, 2080 bp immediately upstream of the *sng-1* ORF), *vha-6* promoter (intestinal expression, 934 bp immediately upstream of the *vha-6* ORF), *col-10* promoter (hypodermal expression, 1126 bp immediately upstream of the *col-10* ORF). All constructs are followed by the *tbb-2* 3'UTR (376 bp immediately downstream of the *tbb-2* stop codon). These constructs were inserted into the pNL43 backbone, which contains a *cbr- unc-119* rescue cassette (Lehrbach & Ruvkun, 2016). The plasmids were used to generate transgenic (miniMos insertion) animals harboring single-copy transgenes (Frokjaer-Jensen et al., 2014). We did not systematically determine the integration sites of the miniMos insertions. We assigned linkage of one of the insertions ( *nicSi744* ) based on segregation away from markers on chromosome V. Plasmids used in this study are listed below.

**Table:** 

Plasmid	Description
pNL549	*rpl-28 p::NbALFA::GFP:: tbb-2*
pNL559	*rpl-28 p::NbALFA::tagRFP:: tbb-2*
pNL594	*myo-3 p::NbALFA::GFP:: tbb-2*
pNL595	*col-10 p::NbALFA::GFP:: tbb-2*
pNL596	*sng-1 p::NbALFA::GFP:: tbb-2*
pNL598	*vha-6 p::NbALFA::GFP:: tbb-2*

*Gene editing by CRISPR/Cas9.*

CRISPR/Cas9 genome editing was carried out through microinjection of guide RNA/Cas9 ribonucleoprotein (RNP) complexes (IDT #1081058, #1072532, and custom crRNA) along with single-stranded oligonucleotides (IDT) as homology-directed repair (HDR) templates (Ghanta & Mello, 2020; Paix et al., 2015). All CRISPR edits were identified by diagnostic PCR and confirmed by sequencing. Guide RNAs and HDR oligos used in this study are listed below.

**Table:** 

Edit created	Guide RNA sequence	HDR oligo sequence
ALFA:: *his-72*	GGTACGAGCCATTGTTGTTC	AttctcaatttccagaacaacaATGCCAT CCCGGCTCGAGGAAGAGCTCCGTCGACGT CTCACCGAGCCAGCTCGTACCAAGCAAAC CGCTCGTAAATCC
ALFA:: *lmn-1*	AGAAAAATGTCATCTCGTAA	taactcttcagaaagcagcgagaaaaATG CCATCCCGGCTCGAGGAAGAGCTCCGTCG ACGTCTCACCGAGCCATCATCTCGTAAAG GTACTCGTAGTTCT
*unc-54* ::ALFA	TAATTGGACTTAGAAGTCAG	CCATCTCGCGCCCGTGCCTCTGACTTCCC ATCCCGGCTCGAGGAAGAGCTCCGTCGAC GTCTCACCGAGTAAgtccaattactcttc aacatccct

*Microscopy*

Fluorescence images were collected on a Leica M165FC equipped with a 910 Leica K5 sCMOS camera using LAS X software (version 3.7.5.24914) or on a Zeiss AxioImager Z1 equipped with an Axiocam 705 CMOS camera and using ZEN software (version 3.3.89.0003). Worms were immobilized using sodium azide and mounted on 2% agarose pads. All images were processed and analyzed using ImageJ/Fiji software (version 2.16.0/1.54p).
